# Recognizing and addressing environmental microaggressions, know-your-place aggression, peer mediocrity, and code-switching in STEMM

**DOI:** 10.3389/feduc.2023.1270567

**Published:** 2023-12-18

**Authors:** Kit Neikirk, Sophielle Silvers, Vijayvardhan Kamalumpundi, Andrea G. Marshall, Estevão Scudese, Melanie McReynolds, Antentor O. Hinton

**Affiliations:** 1 Department of Molecular Physiology and Biophysics, Vanderbilt University, Nashville, TN, United States; 2 Department of Biochemistry and Molecular Biology, Eberly College of Science, Pennsylvania State University, University Park, PA, United States; 3 Roy J. and Lucille A. Carver College of Medicine, Iowa City, IA, United States; 4 Laboratory of Biosciences of Human Motricity (LABIMH) of the Federal University of State of Rio de Janeiro (UNIRIO), Rio de Janeiro, Brazil; 5 Sport Sciences and Exercise Laboratory (LaCEE), Catholic University of Petrópolis (UCP), Petrópolis, Brazil

**Keywords:** know-your-place aggression, environmental microaggressions, white/peer mediocrity, code-switching, diversity equity and inclusion

## Abstract

Diversity, equity, and inclusion (DEI) initiatives are critical for fostering growth, innovation, and collaboration in science, technology, engineering, mathematics, and medicine (STEMM). This article focuses on four key topics that have impacted many Black individuals in STEMM: know-your-place aggression, environmental microaggressions, peer mediocrity, and code-switching. We provide a comprehensive background on these issues, discuss current statistics, and provide references that support their existence, as well as offer solutions to recognize and address these problems in the STEMM which can be expanded to all historically underrepresented individuals.

## Introduction

There are over 19 million individuals^1^ employed in science, technology, engineering, mathematics, and medicine (STEMM) fields, representing over 20% of the US workforce (Okrent and Burke, 2021). Despite being one of the fastest expanding job markets in the US, STEMM consistently lags behind other industries in terms of gender and racial diversity, with underrepresented minorities (URMs) facing various forms of systemic bias and discrimination (Fry et al., 2021). Additionally, URMs are underrepresented in STEMM leadership, accounting for only 28% of full-time faculty positions in STEMM, despite accounting for 32% of the U.S. population^2^.

Broadening participation in STEMM and promoting an equitable distribution of management/leadership roles promises a stronger and more innovative STEMM workforce. However, several institutional and interpersonal barriers exist that make recruitment and retention of URMs in STEMM fields difficult. In this paper, we characterize a few important social barriers such as environmental microaggressions, know-your-place aggression, peer mediocrity, and code-switching. We then present evidence-based, actionable strategies that can be implemented at the institutional level to help break down these barriers for URMs in STEMM.

## Environmental microaggressions

Microaggressions are typically discussed within the context of acts perpetuated by an individual against an individual (Marshall et al., 2021). On the other hand, environmental microaggressions (or systemic microaggressions) are defined as microaggressions that URMs experience as a result of their environment in the form of policies, laws, built environment, and more (Sue et al., 2007). Environmental microaggressions are subtle, often unintentional, acts of discrimination that can create a hostile environment for URMs in higher education, in the laboratory, and the workplace (Sue et al., 2007; McAndrews et al., 2017). These microaggressions can even be amplified among URM with different sexualities (Woodford et al., 2017). Though environmental microaggressions have no individual offenders, they communicate to people who experience them that they are unwelcome and must conform to the majority group to fit in. This racial exclusion can lead to anxiety, depression, and contribute to feelings of isolation (Peterson et al., 2020). A recent qualitative study by Mills (2020) among Black students, many of whom were pursuing STEMM majors, at a predominantly white institution demonstrated that most students experienced segregation, limited representation on campus, and tokenism among several others. Though a small study, these findings among Black students corroborate the lived experiences of URMs in general, who frequently endure the use of gendered language, absence of accessible facilities, tokenism, segregation, and biased hiring practices. Taken together, these serve to greatly disadvantage URM groups in STEMM (Mills, 2020). To date, there are no studies that have documented or characterized the experiences of trainees and faculty from URM groups in STEMM fields specifically. Further qualitative studies are needed so that institutions can characterize how environmental microaggressions are being experienced by URM groups in STEMM fields and address them.

## Know-your-place aggression

Know-your-place aggression has been defined as comments and/or public judgments upon people from URM groups that seek to undermine their achievements and bring about aggression, rather than praise (Mitchell, 2018). This aggression, both literal and figurative, states “*know your place*,” through action and words. Though know-your-place aggression has grown to affect other URM groups, it was originally borne out of historic marginalization, dehumanization, and control of Black people in the United States. A prominent example of know-your-place aggression in recent US history was the #blacklivesmatter protests in 2020 following the murder of George Floyd. Protests were organized in response to differential treatment, brutality, and often unjust incarceration of Black people by police forces. The successful organization of, often peaceful, protests by young people rallying under the movement was usually met with swift and aggressive force, many times at the hands of lay citizens: A symbolic act of know-your-place aggression.

In STEMM, know-your-place aggression is more subtle, and can be difficult to identify. However, when formally studied, it is more commonplace than originally thought. A 2018 study by the National Academy of Sciences revealed that women in STEMM are often targets of know-your-place aggression, with 63% reporting experiences of harassment and discrimination based on race or ethnicity (Johnson et al., 2018). In STEMM, we observe know-your-place aggression manifesting in lay-conversation such as when majority individuals say, “keep your head down until you build a good reputation,” “do not speak in meetings,” and/or “let me take the lead as they know me better.” A major barrier to dismantling know-your-place aggression is the institutionalization of the concept. Black people and several other URM groups are often portrayed by the media as inferior to white races (e.g., promotion of white beauty standards/hair styles, must speak white standard English, etc.) (Liu et al., 2017). Furthermore, URM groups are often not represented in positions of power, further perpetuating know-your-place aggression. Within healthcare, this can manifest in especially sexist ways. While there is roughly an equal number of men and women medical graduates, patients associate men as doctors and prefer them if given a preference (Himmelstein and Sanchez, 2016). We observe women physicians frequently face the “you are a doctor? No, bring the real doctor in.” The subtle promotion of white superiority and lack of representation of URMs in positions of power harm URMs in STEMM and can create a hostile environment. Addressing this takes activism to challenge racial stereotypes and normalize URM thought/culture. Re-evaluation of cultural stereotypes at an institutional level can help make the STEMM workplace a more inviting space for URMs.

## Peer mediocrity

White mediocrity is the idea that society inherently values the voices of white individuals while simultaneously suppressing and greatly undervaluing the voices of URMs (Mitchell, 2018). This is a pervasive problem that is accompanied by a belief that “White is Right” and divergence from this standard signifies a problem, eerily harkening back to white superiority as mentioned earlier. While white mediocrity can occur between White individuals to minority individuals, we believe that this is a pervasive issue where peers, regardless of their identities, can suppress the voices of URMs while promoting themselves despite mediocrity. In STEMM, this frequently dissuades many URMs from accessing upper-level management positions. In fact, a recent report on STEMM workforce diversity by the National Science Foundation revealed that only 28% of full-time faculty positions in STEMM were held by underrepresented minorities, despite accounting for 32% of the U.S. population (See footnote 2). The American Association of Medical Colleges revealed that less than 4% of full-time faculty positions were filled by Black individuals in US allopathic medical schools.^3^ Furthermore, among the healthcare workforce, only 5% of physicians identify as Black.^4^ In the academic sphere, current data suggests that people of color are underrepresented in leadership positions, with only 13% of university department chairs in STEMM being people of color (Gibbs et al., 2016). White applicants are more likely to get hired and promoted due to falsely perceived superiority (Gibbs et al., 2016). While the total number of underrepresented racial/ethnic populations in Dean-level positions at medical schools have doubled since 1993, this number remains at only 12%.^5^ Though a part of the lack of diversity in leadership occurs due to inadequate recruitment efforts, implicit bias in leadership can impair the upward mobility of underrepresented minorities (Neikirk et al., 2023). Beyond this, questioning URM qualifications when novel ideas are presented or selectively ignoring ideas unless presented by a White coworker can feed into know-your-place aggression and fuel the promotion of White individuals (Mitchell, 2018), resulting in a continuous loop of poor recruitment and lack of promotions of URMs at the institution-level.

## Code switching

Code-switching refers to the practice of adjusting one’s behavior, language, or appearance, either consciously or unconsciously, to fit in with a particular group or environment, such as a workplace. It is commonly employed by URMs in professional settings, such as the STEMM field, to navigate and cope with the challenges presented by environmental microaggressions, know-your-place aggression, and peer mediocrity. By adjusting behavior to conform to the dominant culture’s norms and expectations, URMs seek to avoid negative biases, invalidate negative stereotypes, and combat discrimination (Gay, 2018). Code-switching can serve as both a survival strategy for URMs (Brown, 2021) and, in our experience, as a means to gain upward mobility in the workplace. However, it is crucial to acknowledge the physical and psychological toll that code-switching can have on URMs, and address how it can propagate systemic biases and inequities (Rolle et al., 2021). Continuously adapting and suppressing one’s authentic behavior and personality can lead to increased burnout, stress, anxiety, and perpetuate a sense of alienation (Molinsky, 2007). Moreover, the pressure to code-switch may inadvertently reinforce the idea that the dominant culture’s norms and values are superior, further marginalizing URM groups and preserving the vicious cycle of know-your-place aggression and white superiority. Often, professionalism may be used to exclude non-dominant cultural behaviors, however, this can communicate a sense of exclusion for individuals from URM groups. In the context of STEMM culture, this belief can become internalized by URM students, laboratory staff, and other professionals, suggesting that they must set aside their cultural identities when entering the laboratory space.

## Solutions and recognition strategies

At present, URM minorities face remarkable systemic barriers when trying to enter and stay in STEMM fields. These obstacles are exacerbated by discrimination embedded at both institutional and personal levels such as environmental microaggressions, know-your-place aggression, peer mediocrity, and code-switching. To address these challenges, it is crucial for STEMM leadership in both workplace and academic settings to heed the concerns and opinions of URMs with cultural humility and show equal dedication to accommodating their needs. Cultural humility is an important element of the dialogue (Murray et al., 2022). It emphasizes actively striving to learn and respect the beliefs and values of URM individuals and relies heavily on being open to recognizing one’s own cultural limitations and biases. All of this is required in order to help reduce power imbalances in intercultural interactions (Murray et al., 2022). With this in mind, we present a short list of proposed solutions to addressing each of these barriers in Table 1 and elaborate on them below.

Institutions can address environmental microaggressions by offering training on unconscious or implicit biases (Hagiwara et al., 2020) and microaggressions to all employees and ensuring that the work environment is accessible for individuals with disabilities (Crabtree et al., 2022). Perspectives of URM employees should be sought out with cultural humility regarding workplace/academic culture and policies (Fleischmann et al., 2009; Morrison and Grbic, 2015). Environmental microaggressions should also be considered when designing and naming symbols, buildings, or groups. Examples of environmental microaggressions include naming buildings exclusively after White donors or using Native symbols or icons as mascots or names (e.g., Washington Redskins) (Steinfeldt et al., 2018). Thus, surveys for open and anonymous feedback are imperative to allow individuals to have honest conversations about these microaggressions. These surveys must be accompanied by close follow-up from individuals who are willing and able to make actionable changes. Rather than frequently highlighting Christian, White, and Male accomplishments, institutions can increase the representation of URMs by showcasing URM accomplishments and contributions, while emphasizing that, despite systematic barriers, URM individuals are managing to succeed.

## Foster a culture of openness and respect

Leaders can actively promote intentional intercultural dialogues to foster a culture of openness and respect among faculty, staff, and students in institutions of higher learning. Diverse lived experiences and perspectives, especially at the leadership level (Ruiz et al., 2022), must be heard in order to promote an adoption of interventions that reduce barriers for URMs (Fleischmann et al., 2009; Morrison and Grbic, 2015; National Academies of Sciences, Engineering, and Medicine; Division of Behavioral and Social Sciences and Education; Board on Behavioral, Cognitive, and Sensory Sciences; Committee on Advancing Antiracism, Diversity, Equity, and Inclusion in STEM Organizations et al., 2023). These dialogues serve as platforms for meaningful discussions, learning, and celebrating diverse perspectives and backgrounds. They can be organized as roundtable discussions or DEI workshops led by diverse speakers, bringing together individuals from various cultural backgrounds to engage in open and honest conversations about relevant topics, experiences, and challenges to bring into their careers and classrooms (O’Leary et al., 2020). Encouraging participants to share their perspectives fosters empathy and raises awareness of the challenges faced by underrepresented individuals (National Academies of Sciences, Engineering, and Medicine; Division of Behavioral and Social Sciences and Education; Board on Behavioral, Cognitive, and Sensory Sciences; Committee on Advancing Antiracism, Diversity, Equity, and Inclusion in STEM Organizations et al., 2023). Such impactful and respectful dialogues contribute to a more inclusive and understanding community.

## Provide DEI training for all employees

Institutions can effectively address environmental microaggressions by mandating training for all employees on unconscious bias, microaggressions, cultural humility, and cultural sensitivity (Murray et al., 2022). Generally, DEI training is widely supported by employees (Enders et al., 2021), with frameworks to adapt this training for a range of career fields (Dali et al., 2021; Wang et al., 2023). Simply put, DEI training aims to facilitate positive intergroup interaction, and create expectations for inclusive behavior from everyone in the organization to create a positive learning and working environment. Providing uniform training on implicit bias and cultural humility showcases the institution’s dedication to diversity and inclusion (Neikirk et al., 2023), thereby enhancing its reputation among potential employees, students, and partners. To foster inclusivity, institutions should actively seek out perspectives from URM employees, approaching them with cultural humility. This includes considering workplace and academic culture and policies from their unique vantage point.

## Establish employee resource groups

In the absence of or in tandem with DEI offices, employees can initiate changes through the formation of Employee Resource Groups (ERGs). These groups can foster community building, offering mentorship, leadership, and professional development opportunities independently of institutional support (Green, 2018). Past studies have shown that ERGs have tangible effects through reductions in employee turnover (Dutton, 2018) and elevated work engagement via cultivating inclusivity, feelings of connectedness, and a sense of belonging (Cenkci et al., 2019). In the context of STEMM, groups similar to ERGs can be formed including minority writing account ability groups (Spencer et al., 2022), which offer a safe space to write grants and manuscripts. Moreover, leadership should collaborate closely with ERGs and DEI offices to dispel the myth that DEI initiatives are solely for racial and ethnic minorities.

To address both peer mediocrity and code-switching, mentorship, sponsorship, and professional development programs should be established to uplift URM professionals and foster an inclusive and accountable culture. Celebrating the achievements of individuals ensures they are not overlooked in favor of lesser achievements by their well-represented counterparts. Additionally, institutions can allocate funds for hosting employee group activities, encouraging a sense of belonging and engagement within the workplace. Critically, ERGs should encompass both well-represented and underrepresented employees, planning activities, and recognizing talent in an inclusive manner that promotes allyship. Allyship plays a vital role in this solution, as it involves sponsoring URM staff for positions and opportunities (Uddin and De Los Reyes, 2021).

## Incorporate diverse cultural events and celebrations

To foster understanding and appreciation of diverse cultures, institutions can arrange intercultural events and celebrations that highlight various traditions, holidays, and practices (Klak and Martin, 2003; Davis et al., 2023). These events serve to educate the community about different cultures and also provide opportunities for people to come together and celebrate diversity, thus improving student outcomes to appreciate cultural differences (Klak and Martin, 2003). Additionally, there are various organizations dedicated to promoting diversity, equity, and inclusion, such as DEI committees. These groups can host events, workshops, and discussions, amplifying the voices of underrepresented communities and contributing to a more inclusive environment (Davis et al., 2023). While these can be effective for faculty, past frameworks have also established comprehensive workshops for undergraduate students aimed at increasing their retention in graduate school (Marshall et al., 2022a,b,c,d; Barongan et al., 2023). While these workshops are typically aimed at URM students, they also offer a prime avenue to introduce content regarding diverse cultures at the undergraduate level in tandem with existing frameworks for education around mentorship and professional development. This early intervention, paired with regular events for faculty, may increase cultural humility in the long-term while improving the inclusion of individuals from diverse cultures (Marshall et al., 2023).

## Additional strategies

To encourage open dialogue about microaggressions, conducting surveys for anonymous feedback becomes imperative. These surveys should be followed up with actionable change by individuals committed to making a difference. On the other hand, institutions can increase the representation of URMs by showcasing their achievements. Emphasizing URM individuals’ success despite systemic barriers reinforces a more inclusive and supportive environment.

To combat know-your-place aggression, organizations should establish clear policies against discrimination and harassment, DEI and create a safe reporting mechanism for incidents. Additionally, fostering a culture of allyship, where leaders and peers advocate for the rights of persons of URM without being a member of the racial/ethnic group, can help create a supportive environment for URM professionals. At present, DEI offices across the country are being banned or having their funding slashed (Russell-Brown, 2022). This was galvanized, by the 2023 Supreme Court ruling of *Students for Fair Admissions Inc. v. President & Fellows of Harvard College* et al. (Heriot and Kirsanow, 2021) that prevents institutions of higher education to stop considering race in admissions, ostensibly presents a barrier to promoting DEI efforts in higher education and the workplace. This should, however, inspire a shift in responsibility to institutional leadership to assume key DEI roles. This requires holding supervisors and leadership accountable for their actions and implementing mechanisms where they can bring internal and external experts on DEI-related topics as speakers, share resources, and critically evaluate DEI efforts of leadership.

By combatting know-your-place aggressions, organizations can also help to confront peer mediocrity and code-switching. For example, they can create a culture where diversity of thought and background is valued, encouraging employees to bring their authentic selves to work, thus avoiding the necessity of code-switching. In addition to retention, organizations should be more rigorous about their commitment to equitable hiring and promotion practices, such as using diverse hiring panels and implementing a standardized evaluation process. It is important to streamline hiring practices as URM candidates are more likely to leave if they do not hear back within an acceptable time frame.^6^

## Conclusion

Addressing environmental microaggressions, know-your-place aggression, peer mediocrity, and code-switching at the institutional and interpersonal level is crucial to promoting the recruitment and retention of URM in STEMM and promoting DEI. This requires difficult, open-table discussions, where all voices are heard, to lead to institutional change. Here we sought to bring light to these issues, as well as offered tools for implementing strategies to recognize and mitigate their effects on URMs. While many studies have discussed these issues in the context of Black individuals, in our experience that can often affect other URMs, with their prevalence and potential solutions needing further research. We hope that with more widespread recognition of these issues, organizations can create a more inclusive and supportive work environment that empowers all individuals in STEMM to succeed while retaining their unique identities in the process.

## Figures and Tables

**TABLE 1:** Strategies for organizations to create an inclusive and supportive environment where underrepresented individuals feel comfortable being their authentic selves.

1. Foster a culture of openness and respect, by including more intentional intercultural dialogues (e.g., roundtable discussions, DEI lectures given by diverse faculty) where diverse perspectives and backgrounds are valued and celebrated.
2. Provide DEI training for all employees, emphasizing the importance of understanding and appreciating cultural differences.
3. Establish employee resource groups to create safe spaces where underrepresented employees can connect, share experiences, and support each other.
4. Incorporate diverse cultural events and celebrations into the workplace to promote understanding and appreciation of different cultures.

## Data Availability

The original contributions presented in the study are included in the article/supplementary material, further inquiries can be directed to the corresponding author.

## Footnotes

1
https://www.pewresearch.org/science/2021/04/01/stem-jobs-see-uneven-progress-in-increasing-gender-racial-and-ethnic-diversity/

2
https://ncses.nsf.gov/pubs/nsf23315/report

3 see https://www.aamc.org/data-reports/workforce/data/figure-15-percentage-full-time-us-medical-school-faculty-race/ethnicity-2018#:~:text=Diversity%20in%20Medicine%3A%20Facts%20and%20Figures%202019,-Diversity%20in%20Medicine&text=The%20largest%20proportions%20of%20faculty,with%20another%20race%2Fethnicity, accessed July 31st 2023.

4 https://www.aamc.org/data-reports/workforce/data/figure-18-percentage-all-active-physicians-race/ethnicity-2018, accessed July 31st 2023.

5 see https://www.aamc.org/data-reports/faculty-institutions/data/us-medical-school-deans-trends-type-and-race-ethnicity, accessed July 31st 2023.

6 see https://hbr.org/2022/07/its-time-to-streamline-the-hiring-process, Accessed August 1st 2023.
